# Time-On-Task Effects on Working Memory Gating Processes—A Role of Theta Synchronization and the Norepinephrine System

**DOI:** 10.1093/texcom/tgac001

**Published:** 2022-01-13

**Authors:** Shijing Yu, Moritz Mückschel, Sarah Rempel, Tjalf Ziemssen, Christian Beste

**Affiliations:** 1 Cognitive Neurophysiology, Department of Child and Adolescent Psychiatry, Faculty of Medicine, TU Dresden 01309; 2 Faculty of Medicine, University Neuropsychology Centre, TU Dresden 01309; 3 Department of Neurology, Faculty of Medicine, MS Centre, TU Dresden 01309

**Keywords:** EEG, fatigue, norepinephrine system, pupil diameter, time-on-task, working memory

## Abstract

Performance impairment as an effect of prolonged engagement in a specific task is commonly observed. Although this is a well-known effect in everyday life, little is known about how this affects central cognitive functions such as working memory (WM) processes. In the current study, we ask how time-on-task affects WM gating processes and thus processes regulating WM maintenance and updating. To this end, we combined electroencephalography methods and recordings of the pupil diameter as an indirect of the norepinephrine (NE) system activity. Our results showed that only WM gate opening but not closing processes showed time-on-task effects. On the neurophysiological level, this was associated with modulation of dorsolateral prefrontal theta band synchronization processes, which vanished with time-on-task during WM gate opening. Interestingly, also the modulatory pattern of the NE system, as inferred using pupil diameter data, changed. At the beginning, a strong correlation of pupil diameter data and theta band synchronization processes during WM gate opening is observed. This modulatory effect vanished at the end of the experiment. The results show that time-on-task has very specific effects on WM gate opening and closing processes and suggests an important role of NE system in the time-on-task effect on WM gate opening process.

## Introduction

The feeling of fatigue induced by long-time work is prevalent in daily life and is always accompanied by an impaired performance. This phenomenon has been frequently studied using time-on-tasks in laboratory conditions (Lorist et al. 2000; Kato et al. 2009; Möckel et al. 2015) and is often observed in tasks requiring executive functions (Falkenstein et al. 2002; Yu et al. 2021). A likely reason why executive functions or cognitive processes depending on prefrontal cortical structures are affected by fatigue has been outlined in the opportunity cost model by Kurzban et al. (2013) as outlined further below.

When considering executive functions, inhibitory control, cognitive flexibility processes, and working memory (WM) processes (e.g., Miyake et al. 2000; Lehto et al. 2010) are important (Diamond 2013). WM is one of the best-studied cognitive functions in humans. Nevertheless, astonishingly little is known about how time-on-task affects central processes determining WM dynamics—that is, how information supposed to enter WM is controlled or gated. The concept of “WM gating” has been widely used to describe the mechanism of WM flexibly switching between two main functions/states: maintenance and updating (O’Reilly and Frank 2006). When the gate is open, new information can enter WM, and WM information is updated; when the gate is closed, distracting or novel information cannot enter WM, and the stored information is maintained. The dynamics of WM gating processes can be studied using the so-called reference-back paradigm (see Materials and Methods). In this task, gate opening and gate closing can be calculated between different trial types (Kessler and Oberauer 2014; Rac-Lubashevsky and Kessler 2016a). Using the reference-back task, WM opening and closing processes can be measured in various ways, such as response times (Rac-Lubashevsky and Kessler 2016a; Verschooren et al. 2021), event-based eye-blink rate (Rac-Lubashevsky et al. 2017), and neurophysiological measures (Rac-Lubashevsky and Kessler 2018). This is possible because the reference-back task includes comparison trials without requiring the process of WM updating, which, when compared with the classic *n*-back task (Gevins and Cutillo 1993), provides a baseline for comparison and thus enables the identification of the gating processes and calculation of related costs (Rac-Lubashevsky and Kessler 2016a). Higher reaction time (RT) cost was always reported in gate closing than gate opening (Kessler and Oberauer 2014, 2015; Rac-Lubashevsky and Kessler 2016a, 2016b; Rempel et al. 2021). Considering that the gate closed state is a default WM gating mode as suggested by the prefrontal cortex (PFC), basal ganglia working memory (PBWM) model (Hazy et al. 2006), the process of gate closing is a switch from WM demanding status (updating) to default status (maintenance). Therefore, the considerable RT cost in gate closing fits the previous finding that switching to an easier task takes longer than switching to a more difficult task (Gilbert and Shallice 2002; Schneider and Anderson 2010). As opposed to gate closing, gate opening represents a switch from the WM default status (maintenance) to demanding status (updating). Neurophysiological evidence showed that gate opening, but not gate closing, was associated with strong basal ganglia, thalamic, and fronto-parietal activations (Nir-Cohen et al. 2020). This suggests, as a selective process driven by specific stimulus, gate opening requires more intentional control than gate closing. This is of particular relevance considering time-on-task effects. According to the before-mentioned account by Kurzban et al. (2013), the costs of performing a task are represented while performing a task and increase during time-on-task. Costs are exceptionally high when cognitive operations require intentional control. Moreover, WM gating functions require task-switching and cognitive flexibility processes (Kessler and Oberauer 2014; Rac-Lubashevsky and Kessler 2016a), which have recently been shown to indicate strong time-on-task effects (Yu et al. 2021). Therefore, it is reasonable to hypothesize that time-on-task effects are stronger during WM gate opening processes than WM gate closing processes. We examine this hypothesis with particular emphasis on neurophysiological processes.

Regarding neurophysiological processes, we primarily focus on theta-band dynamics. Theta oscillations play a primary role in WM control (Klimesch 1999; Başar et al. 2001; Sauseng et al. 2010; Karakaş 2020) and in its orchestration with cognitive control and response selection processes (Chmielewski et al. 2016, 2017; Dippel et al. 2017; Adelhöfer and Beste 2020; Takacs et al. 2020). Importantly, theta band activity (TBA) seems to be particularly relevant during the sequential encoding of WM items (Roux and Uhlhaas 2014) for which computational models suggest that input-gating mechanisms regulate these dynamics (Chatham and Badre 2015; Rac-Lubashevsky and Kessler 2016a). We assume that TBA during gate opening is expected to decrease with time-on-task. Using electroencephalography (EEG)-beamforming methods (Gross et al. 2001), we delineate which functional neuroanatomical structures are associated with theta band time-on-task effect during WM gate opening. Here, we expect prefrontal regions to show modulations because the regions are particularly prone to time-on-task effects (Kurzban et al. 2013; Yu et al. 2021), playing an essential role in WM processes and WM gate opening in particular (Nir-Cohen et al. 2020). However, several lines of evidence suggest that TBA during cognitive control encodes multiple levels of information; that is, information about the stimulus being presented, information on how to respond to a stimulus, and information specifying the motor process itself (Chmielewski et al. 2017; Dippel et al. 2017; Mückschel et al. 2017; Giller et al. 2020; Pscherer et al. 2020). These insights were made possible by applying residue iteration decomposition (RIDE) (Ouyang et al. 2011, 2015) on single-trial EEG data, time-frequency-transformed after that. RIDE yields three functionally distinct activity clusters: 1) the S-cluster captures perceptual and attentional selection mechanisms, 2) the C-cluster contains information specifying how to map a stimulus on the appropriate response, and 3) the R-cluster reflects processes of the motor execution. In principle, all of this information is central to control during WM gating processes. Therefore, all of these TBA clusters are likely to show time-on-task effects. However, according to the model by Kurzban et al. (2013), especially effortful decision processes depending on prefrontal structures are prone to fatigue or time-on-task effects. Since these processes are reflected by the C-cluster (Ouyang et al. 2011, 2015, 2017), it is possible that especially C-cluster TBA shows time-on-task-effects.

Based on this assumption, we were interested in probable modulatory processes associated with the time-on-task effects on the WM gating, especially on gate opening. WM strongly depends on the PFC, where various neurotransmitters modulate WM (Motley 2018). Among these neuro-modulators, norepinephrine (NE) has been suggested to strongly impact WM functions in the PFC (Arnsten et al. 1996; Zhang et al. 2013). Specifically, NE within the PFC exerts an inverted-U-shaped modulation of WM performance. Moderate NE levels promote WM performance by decreasing distractibility. Low or exaggerated NE levels impair WM performance (Arnsten et al. 1996; Robbins and Arnsten 2009). This inverted-U-shaped modulating function of the NE was also described in the adaptive gain theory combining two NE modes: phasic and tonic modes (Aston-Jones and Cohen 2005). It was suggested that particularly the phasic mode of the NE system is driven by task-related decision processes, and a strong phasic NE response indicates high task engagement (Aston-Jones and Cohen 2005). Phasic NE arousal was observed to amplify perception and memory (Mather et al. 2016) and attentional performance (Howells et al. 2012). All these processes are necessary for gate opening as a stimulus-driven process. According to this evidence, gate opening, which demands high PFC control on inhibiting distracting information and attention switching, is likely modulated by phasic NE activities. By contrast, gate closing requires less cognitive control and is, thus, less modulated by phasic NE activities. In this study, we record pupil diameter data as a representation for NE release. Evidence shows that the pupil diameter covaries with the NE system and is used as a reliable indicator of NE activities in many studies (Hou et al. 2005; Gilzenrat et al. 2010; [Bibr ref31][Bibr ref31]; Murphy et al. 2011; Hong et al. 2014; Hopstaken et al. 2015). Mainly, baseline-corrected pupil size represented phasic NE activities (Gabay et al. 2011; Joshi et al. 2016; Reimer et al. 2016; Wolff et al. 2018).

To examine the modulatory role of the NE system in WM gate opening in the context of time-on-task effects, we correlate the time series of phasic pupil diameter and task-related theta activity in PFC. The interaction between pupil diameter and task-related theta activity is expected to exhibit at a high level at the beginning of the experiment, showing a strong NE modulation effect. However, the modulatory effects of phasic NE activities are unlikely to remain at a consistently high level in WM gate opening according to frequently observed decrease of phasic NE activities in time-on-task indicating task disengagement (Hopstaken et al. 2015). According to the opportunity cost model, the increase of opportunity cost/effort of high-demanding WM gate opening in time-on-task reduces engagement in the primary task and increases engagement in task alternatives (Kurzban et al. 2013). In line with the adaptive gain theory, the neural correlates of the assumed performance decline of the WM gate opening (expectedly at the end of the experiment) might be related to the decrease of phasic NE activities that the modulatory effect phasic NE activities may diminish accordingly. Thus, the control-related activities in the PFC were merely driven by the NE system. To conclude, we expected a time-on-task effect, particularly on the WM gate opening, and this time-on-task effect could be observed through the existence of a strong correlation between the phasic pupil diameter and the task-related prefrontal theta activity at the beginning of the experiment and the decrease/disappearance of the correlation at the end of the experiment.

## Materials and Methods

### Participants

The *n* = 38 healthy volunteers (13 male, mean age: 25.24 ± 2.84) participated in the experiment. Among them, *n* = 31 participants (12 male, mean age: 25.74 ± 2.53, all right-handed) completed the experiment and were included for data analysis. All participants had a normal or corrected-to-normal vision. They were required to consume no caffeine beverages in the morning before the experiment, which started around 9 AM. All participants provided written informed content before the experiment and were reimbursed with 35 euros after the experiment. The Ethics Committee of the Medical faculty of the TU Dresden approved our study, and the experiment was conducted following the Declaration of Helsinki.

### Task and Procedures

**Figure 1:**
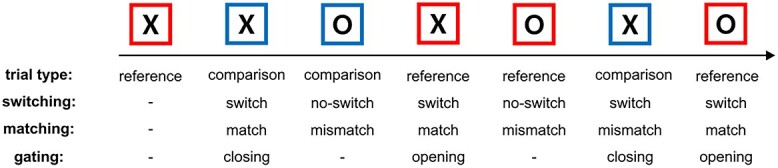
An example of the reference-back task and the description of the stimulus. The arrow indicates the order of stimulus presentation.

We adapted the reference-back paradigm (Rac-Lubashevsky and Kessler 2016a) to a time-on-task. A capital letter (“X” or “O”) framed by a colored square (in blue or red) was presented in each trial. Participants were required to decide whether the presented letter was identical to the red-framed letter displayed previously. The right “Ctrl” button should be pressed when the letters are identical, and the left “Ctrl” button if the letter is not identical. The reference-back task required participants to consistently store the previously red-framed letter in WM and update it when a new red-framed letter showed up. Accordingly, trials with red-framed letters were reference trials, and the ones with blue-frames were letters in comparison trials, that is, they were only used for comparison with the previous red-framed letter. Trials with a frame in the same color as the previous trial were no-switch trials, while the ones with a frame in a different color as the previous one were switch trials. Moreover, the required response differentiated trials into match (identical) trials and mismatch (not identical) trials. An example of the reference task and the trial definition is presented in Figure 1.

Each trial started with a fixation cross presented for 600–1000 ms, followed by the stimulus presentation for 1500 ms or till a response was made. The screen turned blank for 1000 ms afterward. Before the experiment, a detailed introduction and a 30-trial exercise were provided to familiarize participants with the task. The formal experiment consisted of 3600 trials and took around 2.5 h. Thirty percent of the trials were switch trials. The frame color and the required response were assigned in a balanced manner. The order of stimulus presentation was randomized but was the same for all participants. To measure the pupil size in resting status, a 1-min fixation period was assigned before and after the experiment, and a 10-s fixation period was assigned every 180 trials during the experiment. Participants were required to stare at the fixation cross in the display center without any reaction during the fixation period. Previous studies (Lim et al. 2016; Yu et al. 2021) showed that short breaks around 10 s did not affect time-on-task effects. No more extended break was included during the entire experiment to avoid cognitive performance recovering from a break lasting for several minutes (Möckel et al. 2015). The starting trial after each fixation period was in a red frame and was not counted in the 3600 trials.

### Computation of Gating Indices

All trials (*n* = 3600) were equally divided into four sessions, with 900 trials each for each participant. In each session, trials were categorized into eight conditions according to three features: reference/comparison, switch/no-switch, and match/mismatch. In each session, gate opening and gate closing indices were calculated using trials with correct responses according to the previous study of Rac-Lubashevsky and Kessler (2016a) as given in formulas 1 and 2, respectively:

“Gate opening = (switch_match_reference + switch_match_reference)—(no-switch_match_reference + no-switch_mismatch_reference)” Formula 1.

“Gate closing = (switch_match_comparison + switch_match_comparison)—(no-switch_match_comparison + no-switch_mismatch_comparison)” Formula 2.

As shown in the formulas, gate opening is calculated as the switching cost of reference trials, while gate closing is the switching cost of comparison trials. In reference trials, participants were required to update the reference letter; in comparison trials, participants needed to maintain the previous reference. Accordingly, the switching processes in WM gating were defined in terms of updating and maintenance. Switch reference trials represent a change from close to open status of WM gate, while no-switch reference trials represent consistent open status of WM gate and can be seen as a baseline of the updating process. Therefore, the contrast between them describes the action of opening the gate. Following the same logic, the contrast between switch comparison trials and no-switch comparison trials represents the gate closing action which turns the active reference updating process to a default WM status of maintenance.

### Pupil Diameter Recording and Processing

Pupil diameter data were recorded by a RED 500 eye tracker using the software iView X (SensoMotoric Instruments GmbH) at a sampling rate of 256 Hz. The eye-tracker was attached underneath the display around 60 cm away from the participants. After the recording, raw pupil diameter data were synchronized with EEG data from the same participant according to the identical start and end markers in both datasets using the EYE-EEG extension of EEGLab (http://www2.hu-berlin.de/eyetracking-eeg/). After that, high-frequency activities were removed by a low-pass filter of 20 Hz, and a median filter removed pupil spikes. Artifacts, such as eye movements, were linearly interpolated using an open-source toolbox developed by Kret and Sjak-Shie (2019). After preprocessing, pupil diameter data from both eyes were averaged for each participant. Pupil diameters during the resting status were segmented according to corresponding markers and were averaged across time for each fixation period. Task-related pupil diameters were divided into four sessions, and 900 trials were segmented for each session according to the stimulus markers. Each trial started from 1000 ms before stimulus onset to 2000 ms after stimulus presentation. All trials in each session with correct responses were categorized into eight conditions as outlined in the “Computation of Gating Indices.” For each condition and session, task-related pupil diameter was averaged across all “corresponding” trials and were baseline-normalized using the averaged pupil diameter between −200 and 0 ms relative to stimulus onset.

### E‌EG Recording and Processing

The EEG was recorded from 60 equidistantly positioned Ag/AgCl electrodes. The coordinates of ground and reference electrodes were theta = 58, phi = 78, and theta = 90, phi = 90, respectively. EEG data were recorded simultaneously with the pupil data recording during the experiment using BrainVision Recorder software package (Brain Products, Inc.) with a sampling rate of 500 Hz. After recording, the raw EEG data were preprocessed in BrainVision Analyzer 2 software package (Brain Products, Inc.) with the following steps. First, the EEG signals were down-sampled to 256 Hz. Then, infinite impulse response filters from 0.5 to 40 Hz at a slope of 48 dB/oct, and an additional notch filter of 50 Hz was applied. After that, we discarded the defective electrode channels and applied a new reference calculated from the remaining channels. Furthermore, the regular artifacts, such as eye movements and pulses, were removed by independent component analysis (infomax algorithm), and irregular artifacts such as technical noises were removed via a manual raw data inspection. Subsequently, previously discarded channels were interpolated by spherical spines using neighboring electrodes. After EEG preprocessing, the continuous EEG data were segmented into single trials for four sessions as in pupil diameter data. The time length of each trial was 4000 ms, with 1000 ms before the stimulus and 3000 ms after the stimulus. After the stimulus, a long time window was set to avoid the edge effects in further time-frequency decomposition processes. For each trial, an automatic artifact rejection was processed to remove the residual artifacts with the following criteria: a maximal value difference above 150 μV in an interval of 200 ms, minimal amplitude <−100 μV or maximal amplitude >100 μV, or an activity (max-min) <0.5 μV in an interval of 200 ms. All trials in each session with correct responses and without artifacts were categorized into eight conditions as described in the Computation of Gating Indices section.

### Residue Iteration Decomposition

The RIDE was implemented with the “RIDE toolbox” developed by Ouyang et al. (2011) using segmented single-trial EEG data. Baseline correction was applied on each trial from −200 to 0 ms relative to stimulus presentation before RIDE. For each session and condition defined previously, three RIDE clusters were decomposed for every single trial: an S-cluster related to stimulus-related processes, a C-cluster representing central activities of stimulus–response translation, and an R-cluster related to motor response execution. We used the following time windows to calculate the S-, C-, and R-clusters: S-cluster: −200 to 900 ms relative to stimulus onset, C-cluster: 200–900 ms relative to stimulus onset, and R-cluster: −300 to 300 ms relative to response, respectively. The C-cluster latencies are iteratively updated by applying L1-norm minimization during RIDE. More details about RIDE-cluster computation can be found in previous publications (Ouyang et al. 2015). Each trial was decomposed into three clusters with the same length as in the original trial (i.e., 4000 ms starting from 1000 ms before the stimulus and 2000 ms after the stimulus).

### Time-Frequency Decomposition

For each RIDE cluster in each session and condition, we decomposed the time-frequency representations of theta oscillations (4~7 Hz) using the FieldTrip toolbox and wavelet time-frequency transformation (Oostenveld et al. 2010). Morlet wavelets in the time domain for theta frequency in a step of 0.5 Hz were calculated, where the wavelet duration was three and the number of wavelet cycles was 5.5. After that, the time-frequency representation of theta oscillations was normalized by baseline activities between −200 and 0 ms relative to stimulus onset; that is, a decibel conversion calculated as *P*_dB_ = 10 × log_10_(*P*_toi_/*P*_baseline_) (*P* is power, and toi refers to “time of interest”) was performed. For each participant and session, baseline-normalized theta powers for gate opening and gate closing were calculated following Formulae 1 and 2 (see above). To examine when the time-on effects (i.e., the difference between the first and the last sessions) were observed in gate opening and gate closing theta activities, cluster-based permutation tests comparing sessions S1 and S4 using time-frequency representations were applied for gate opening and closing separately. This step revealed a significant difference between session S1 and S4 around 0.5–1.5 s for all RIDE clusters and only for the gate opening condition (see Results). Hence, further analyses were based on theta powers between 0.5 and 1.5 s only for gate opening theta power. We applied cluster-based permutation tests to identify the electrodes showing significant difference between sessions S1 and S4 in this time window to the averaged frequency representation between 0.5 and 1.5 s of each RIDE cluster. All cluster-based permutation tests were based on the dependent *t*-tests on each electrode (and time points). The Monte-Carlo method was used to compute the reference distribution of the permutation test with 500 random draws. The threshold for the sample-specific *t*-tests was 0.05. The cluster-level *t*-values were computed using the sum of all *t*-values within electrodes (and time points). The minimum number of electrodes (or time points) forming a cluster was 1.

### Source Estimation

The neuroanatomical source activities showing the S1 − S4 difference of task-related theta band activities were estimated using dynamic imaging of coherent sources beamformer (Gross et al. 2001) for each RIDE-cluster only for gate opening condition according to the cluster-based permutation tests using sensor-level data. For each participant, theta activities of gate opening in sessions S1 and S4 were selected between 0.5 and 1.5 s after stimulus presentation. The corresponding baseline theta activities were selected from −1 to 0 s relative to stimulus onset for the abovementioned trials. Individual theta frequency power and the cross-spectral density matrix were calculated for these conditions using a single Hanning taper frequency transformation. After that, a spatial filter was constructed using all baseline and activity conditions with a regulation parameter of 5%. We took the same number of trials in all conditions to construct the spatial filter to avoid spurious noise-related sources (Handy 2009) (determined by the condition with the least number of trials). The trials in each condition used for constructing the spatial filter were randomly selected. This spatial filter was further applied to the individual power to estimate the source. Afterward, the source power of each session and condition was baselined-normalized in decibel as *P*_dB_ = 10 × log_10_(*P*_toi_/*P*_baseline_) (*P* is power). Based on the decibel power of each condition, the source power of gate opening for each session was calculated following Formula 1. The average gate opening source theta powers were then computed by averaging across participants for each session and were mapped on FieldTrip head model template “standard_mri.” After that, we selected the top 1% voxels showing positive S1 − S4 difference to construct the neuroanatomical clusters of interest using the “DBSCAN” algorithm. The minimum number of voxels to form a cluster was seven. We then reconstructed the activities in the region of interest through a linearly constrained minimum variance beamformer (Van Veen et al. 1997). This was conducted in each anatomical cluster for corresponding RIDE clusters. For the gate opening condition and each participant and session, a covariance matrix was computed using corresponding RIDE-decomposed single trials to generate a spatial filter which was then applied on the RIDE-decomposed data to reconstruct the time series of each corresponding source indices. The time series was then averaged across employed indices and was further time-frequency-decomposed using Morlet wavelets as sensor-level time-frequency decomposition. Time-frequency representations of gate opening task-related theta powers were calculated in the same way as in the previous steps.

### Statistical Analysis

The statistical analysis on behavioral data utilized two parameters: accuracy and RT calculated for each session, condition, and participant. RT data were only calculated from trials with correct responses. To examine the time-on effects on behavioral performance, repeated measures ANOVAs using within-subject factor “session” (S1, S2, S3, and S4) were applied on accuracy and RT parameters for each condition. After that, the accuracy and RTs of gate opening and closing were computed separately using Formulas 1 and 2 for each condition and participant. Repeated measures ANOVA using within-subject factors “session” (S1, S2, S3, and S4) and “gating” (opening and closing) were calculated for RT and accuracy data.

**Figure 2:**
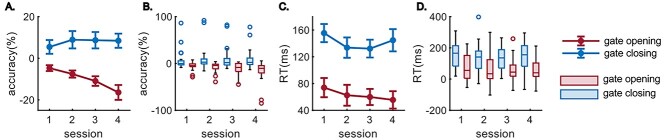
Behavioral performance of the reference-back task. (*A*) and (*B*) reveal the line plot and boxplot of the same data, showing the gating effects (opening and closing) on accuracies. (*C*) and (*D*) are the line plot and boxplot showing the gating effects on RTs. For all line plots, error bars indicate the standard error of the mean. In boxplots, the central line indicates the median, and the bottom and top edges of the box indicate the 25th and 75th percentiles, respectively. Each dot represents an outlier defined by Matlab “boxchart” function.

The statistical analyses were separately conducted on task-related pupil activities and resting status pupil baselines for pupil diameter data. For the task-related pupil diameter, gate opening and closing data were calculated for each session using Formulas 1 and 2. Paired-samples *t*-tests were separately applied on gate opening and closing pupil diameter for each time point from 0 to 2 s after stimulus presentation comparing sessions S1 and S4. For pupil baselines during the fixation periods, a paired-sample *t*-test was applied to compare the averaged pupil baselines between the start and end, and repeated measures ANOVA was applied on pupil baselines during the experiment, respectively.

For all repeated measures ANOVAs, Greenhouse–Geisser correction was applied when necessary and post hoc tests were Bonferroni-corrected.

To examine the possible interaction between NE dynamics and cortical TBA and to investigate how their interaction change with time/sessions, the correlation between task-related pupil diameters and source-level theta activities was conducted for each RIDE cluster in each anatomical cluster. The correlation analysis was applied only on gate opening conditions for sessions S1 and S4 because time-on-task effect was only observed in the behavioral performance of gate opening. Task-related source-level theta activities were selected from 0 to 1.5 s, and baseline-normalized pupil diameter was selected from 0 to 2 s.

## Results

### Behavioral Performance

The behavioral data are presented in Figure 2. The repeated measures ANOVA using accuracies revealed a main effect of “session” (*F*(2.16, 64.78) = 4.01, *P* = 0.020, η^2^ = 0.12), showing a general decline in the gating effect from session S1 (4%) to S4 (−4%). The main effect gating was also significant (*F*(1, 30) = 11.52, *P* = 0.002, η^2^ = 0.28), and the accuracy of gate opening (−9.9%) was lower than for gate closing (7.9%). The interaction between factors “session” and “gating” was also significant (*F*(2.3, 69.71) = 7.09, *P* < 0.001, η^2^ = 0.19). The post hoc repeated measures ANOVA comparing gate opening effects in all sessions revealed a significant effect of “session” (*F*(1.81, 54.16) = 9.35, *P* < 0.001, η^2^ = 0.24) with a decreasing trend from session S1 (−4%) to S4 (−16.4%). However, no effect of “session” was found using gate closing data (*F*(2.82, 84.73) = 1.19, *P* = 0.319, η^2^ = 0.04). The repeated measures ANOVA using RTs showed a main effect of “gating” (*F*(1, 30) = 25.09, *P* < 0.001, η^2^ = 0.46). The RT of gate opening (63 ms) was smaller than for gate closing (142 ms). No other effects were observed (all *F* ≤ 1.50, *P* ≥ 0.23, η^2^ ≤ 0.05).

### Task-Related Theta Activities

**Figure 3:**
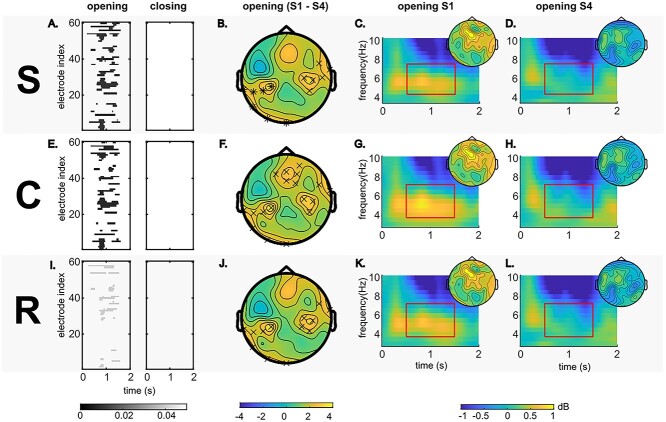
Task-related theta activities at the sensor level. Plot (*A*) shows the electrodes and the time points of the difference of gating effects between sessions S1 and S4 for the RIDE S-cluster data. Only the data points with significant differences (*P* ≤ 0.05) are presented. The color bar indicates the *P* value. Plot (*B*) shows the topography of the theta power difference of gate opening between sessions S1 and S4 from 0.5 to 1.5 s for the RIDE S-cluster data. The color bar shows the *t*-values. “×” and “*” represent the significance of *P* ≤ 0.05 and *P* ≤ 0.01, respectively. Plots (*C*) and (*D*) present the time-frequency decomposition of gate opening theta oscillations of RIDE S-cluster using electrodes with significant S1-S4 difference as in plot (*B*) for sessions S1 and S4, respectively. Theta frequency and the time of the interest (0.5–1.5 s) are marked by a red rectangle. The topography of the task-related gate opening theta power between 0.5 and 1.5 s for each session was presented on the upper-right side. The color bar indicates the task-related theta power in dB. Plots (*E*–*H*) and plots (*I*–*L*) correspond to the descriptions of plots (*A*–*D*) for RIDE-C and RIDE R-clusters, respectively.

The RIDE-decomposed theta activities in the sensor level are shown in Figure 3. For all RIDE clusters (S, C, and R), significant differences (*P* ≤ 0.05) between sessions S1 and S4 were observed for gate opening but not for gate closing (Fig. 3*A*,*E*,*I*). The time windows showing significant differences of task-related gate opening theta powers were centered around 1 s for all RIDE clusters. Therefore, we selected gate opening task-related theta powers between 0.5 and 1.5 s for further analyses. From 0.5 to 1.5 s after stimulus presentation, a significant difference between sessions S1 and S4 was observed at bilateral electrode sites for all RIDE clusters and was also observed at frontal sites for the C-cluster. The time-frequency representations of task-related gate opening theta activities at the electrode sites showing significant differences for each RIDE cluster (Figs 3*C*,*D*,*G*,*H*,*L*) indicate that the gate opening effects were quite strong in session S1 but negligible in session S4.

**Figure 4:**
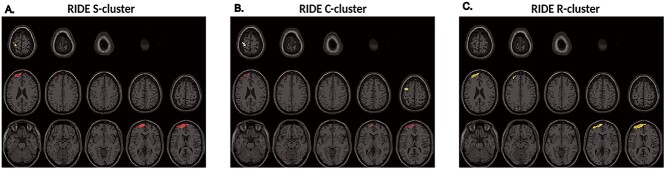
Anatomical sources of gate opening effects. Plots (*A*–*C*) show the voxels of the top 1% positive S1 − S4 difference of task-related gate opening theta activities using RIDE-clusters S, C, and R, respectively. The color in each plot indicates an anatomical cluster.

**Figure 5:**
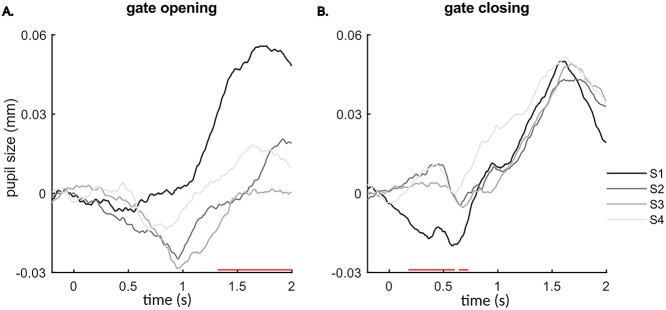
Pupil diameter results. (*A*) and (*B*) present the pupil diameter changes of gate opening and closing, respectively. Red lines indicate the time points of significant difference between sessions S1 and S4 (*P* ≤ 0.05).

The anatomical regions of the highest positive S1 − S4 difference of task-related gate opening theta powers are presented in Figure 4. For the RIDE S-cluster, the voxels formed two anatomical clusters showing the highest positive S1 − S4 difference (Fig. 4*A*). The largest anatomical cluster (S-source 1) was located in the left dorsolateral, medial, and orbital superior frontal gyrus (BA9, 10, and 46) and extended to the left middle frontal gyrus (BA46). A relatively small cluster (S-source 2) was also observed in the left postcentral gyrus (BA3, 1, and 2). For the RIDE C-cluster, three anatomical clusters were evident (Fig. 5*B*). The largest anatomical cluster (C-source 1) was left-lateralized in the dorsolateral and medial superior frontal gyrus (BA9 and 46) and the middle frontal gyrus (BA46). The other clusters (C-source 2) were mainly located in the left precentral and postcentral gyri (BA4, 3, 1, and 2) and extended to the left middle frontal gyrus (BA46). For the RIDE R-cluster (Fig. 4*C*), the difference was centered in one anatomical cluster (R-source 1), which was located in the left dorsolateral and medial superior frontal gyrus (BA9 and 46) and the left middle frontal gyrus (BA46).

### Pupil Diameter

Figure 5*A* and *B* represented the baseline-corrected pupil diameter of gate opening and closing, respectively. A significant difference between the first and the last sessions (S1 and S4) was observed between 1.32 and 2 s for gate opening, showing a larger pupil dilation in the first session and between 0.18 s and 0.73 s for the first session gate closing that pupil size in session S4 was larger than in S1. The baseline-normalized pupil dilation in each condition and pupil sizes during the resting times are presented in the Supplementary Figure S2. Behavioral performance for each condition is shown in Supplementary Figure S1.

### Correlation between Source-Level Theta Activities and Pupil Diameter

**Figure 6:**
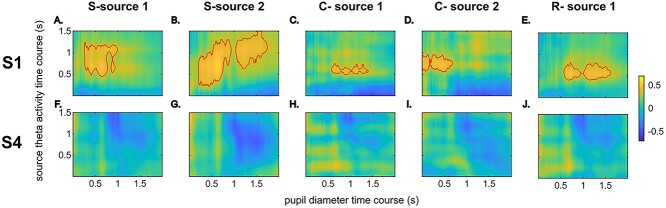
Correlations between source-level gate opening task-related theta activities and pupil activities. Plots (*A*–*E*) show the correlation in session S1. Plots (*F*–*J*) are the corresponding correlation matrix in session S4 for plots (*A*–*E*). The respective title indicates the RIDE cluster and the anatomical cluster of the source theta activities. The index of anatomical sources was defined in the Results section, “Task-related theta activities.” Red boundaries mark only positive correlation clusters formed by over 5000 data points.

The correlations between source-level theta activities and pupil activities in each RIDE cluster in respective anatomical clusters are illustrated in Figure 6. Significant positive correlations were observed for all RIDE clusters in all anatomical sources in the first session (S1) but not in the last session (S4).

In session S1, the source-level task-related theta power of RIDE S-cluster in the frontal cortex (i.e., S-source 1) around 0.5–1.2 s was correlated with baseline-normalized pupil diameter around 0.3–1 s (46 738 data points, mean *R* = 0.40, and mean *P* = 0.029). Two large correlation clusters were observed for the source-level task-related theta power of the RIDE S-cluster in the precentral and postcentral gyri (i.e., S-source 2) in session S1. One was around 0.2–1.3 s for theta activities and around 0.2–1 s for pupil activities (61 789 data points, mean *R* = 0.43, and mean *P* = 0.020). Another one was evident around 0.6–1.5 s for theta activities and around 1–2 s for pupil activities (46 517 data points, mean *R* = 0.40, and mean *P* = 0.028).

For the RIDE C-cluster in session S1, a strong positive correlation was observed between the source-level theta activities in the frontal cortex (i.e., C-source 1) around 0.5–0.8 s and pupil activities around 0.5–1.5 s (12 821 data points, mean *R* = 0.38, mean *P* = 0.035). The correlations between the source theta activities of C-source 2 and phasic pupil diameter were also significant, and they formed two adjacent correlation clusters. Together, these two correlation clusters were located around 0.5–1 s for task-related theta activity and around 0–0.7 s for pupil diameter (23 030 data points, mean *R* = 0.41, and mean *P* = 0.024). For the RIDE R-cluster in session S1, the correlation between its source theta power in the frontal regions (R-source 1) and pupil activities was observed between 0.4 and 0.7 s for theta activities and between 0.5 and 1.6 for pupil activities (32 464 data points, mean *R* = 0.41, and mean *P* = 0.027). In session S4, no significant positive correlation clusters with over 5000 data points were observed.

## Discussion

The primary goal in the present study was to examine the time-on-task effects on WM gating functions (i.e., gate opening and closing), including its neurophysiological basis and functional neuroanatomy. To achieve this, we utilized a reference-back paradigm in a time-on-task setting, recorded EEG signals, and tracked the pupil diameter as indirect measures for NE system dynamics. This was based on our assumption that the NE system may modulate the time-on-task effects on WM gating functions. To examine which subprocesses of WM gating functions were prone to time-on-task effect, the RIDE method was employed to distinguish cognitive subprocesses relevant to stimulus, response, and the transitional processes between stimulus evaluation and responding. We also applied beamforming techniques to extract the anatomical source of the time-on-task effect for each subprocess. The results revealed an evident time-on-task effect. There were time-on-task effects for WM gate opening but not on WM gate closing.

Corroborating previous studies, the switching costs in RT during WM gate closing were higher than in WM gate opening throughout the entire experiment, reflecting the different cognitive processes of gate opening and closing (Kessler and Oberauer 2014, 2015; Rac-Lubashevsky and Kessler 2016a, 2016b; Rempel et al. 2021). The difference of RT costs between gate opening and closing fits the PBWM model that WM gate opening is a more active process than gate closing (Hazy et al. 2006). Though rare studies used the switching cost of accuracy as the indicator of the WM gating, our study demonstrated a consistent accuracy cost in gate opening but not in gate closing. This corroborates that WM gate opening was a more difficult task than WM gate closing.

The most important finding of the behavioral data was that the switching cost in the accuracy data increased with time during gate opening, while no significant effects were obtained during gate closing. This dissociation shows that WM gate opening but not closing processes are affected by time-on-task effects. This dissociation of time-on-task effects between gate opening and closing is also reflected in theta band dynamics showing effects during WM gate opening but no significant effects during WM gate closing. Time-on-task effects were seen for all of the isolated RIDE clusters, which suggests that all aspects of information coded in the theta signal are affected by time-on-task effects during WM gate opening processes. This result is reasonable considering that all information about stimulus identity, stimulus–response relations, and the motor response is essential for goal-directed behavior (Rac-Lubashevsky and Kessler 2016b). For gate opening, the difference of task-related theta activities between sessions S1 and S4 was mainly observed between 0.5 and 1.5 s after stimulus onset, starting when the response was executed (i.e., between 457 and 692 ms) and ending before the subsequent trial. In this time window, the task-related theta synchronization during WM gate opening in session S1 was particularly strong at frontal electrodes sites. Thus, there was a robust theta synchronization at the beginning of the experiment where gate opening processes were most efficient, as indicated by the behavioral data. Previous studies suggested that theta synchronization processes are essential during the encoding and retrieval of contextual information (Klimesch et al. 1997, 2001). Especially in the PFC, theta synchronization processes promote WM performance (Benchenane et al. 2011; Alekseichuk et al. 2016). Interestingly, the results in the beamforming analysis revealed that differences in the degree of theta synchronization processes between sessions S1 and S4 were associated with the left dorsolateral prefrontal cortex (DLPFC). The decrease in theta synchronization in this region can explain emerging difficulties in WM gate opening processes as reflected at the behavioral level. The DLPFC plays an essential role in context updating for cognitive control (O’Reilly 2006; O’Reilly and Frank 2006; Badre 2012; Nee and Brown 2013). A few studies interpreted the activation of PFC after response as a process of refreshing just-activated representation for prospective utilization (Johnson et al. 2005; Raye et al. 2007). Thus, at the beginning of the experiment, the highly activated theta synchronization in the DLPFC might indicate the strong control of WM gate opening to guarantee a successful updating process. These specific WM processes are then affected by time-on-task. According to the opportunity cost model (Kurzban et al. 2013), executive functions in PFC are prone to time-on-task effects. The decline of theta synchronization in the PFC suggests an impaired WM gate opening processes, leading to the increased error rate in gate opening performance. The results corroborate predictions of the opportunity cost model in terms of functional neuroanatomical predictions. However, the data also qualify the opportunity cost model by showing that only specific prefrontal cortical functions (i.e., WM gate opening processes) are affected by time-on-task. However, besides the DLPFC effects in theta synchronization processes between sessions S1 and S4, the pre- and postcentral gyri for RIDE S- and C-clusters represented stimulus-related process and transitional process between stimulus and stimulus and response, respectively (Ouyang et al. 2011). The pre- and postcentral gyri is part of the motor-somatosensory cortical network associated with sensory and motor processing (Bigbee 2011). Altogether, the high task-related theta activity in the first session suggests an active “encoding” processes of reference stimulus in WM gate opening, and its decline shows that the time-on-task effect also impaired the “encoding” process of a new reference.

Most importantly, task-related theta band effects during gate opening were strongly correlated with phasic pupil dynamics in the first session, but these significant correlations vanished in the last session. In the first session, the task-related theta oscillations were activated at a relatively high level than in the last session. Meantime, the phasic pupil amplitude in gate opening was also evident, indicating a strong phasic NE activation relevant to the task engagement and mental effort invested (Beatty 1982; Aston-Jones and Cohen 2005; Gilzenrat et al. 2010; Eckstein et al. 2017; van der Wel and van Steenbergen 2018; da Silva et al. 2021) in WM gate opening. When closing the gate, no difference of phasic pupil peaks between sessions S1 and S4 was also observed, suggesting the allocated mental effort stayed at the same level. Likely, the phasic NE activity in the first session plays an essential role in the PFC gate opening functions, as suggested by the strong positive correlations between pupil diameter and theta band dynamics at the source level. These correlation matrices in the session S1 appeared relatively early for phasic pupil activation and late for theta synchronization, suggesting that the high NE activation likely modulated gate opening-related processes in cortical regions. This early NE modulation might be driven by novel reference information (Foote et al. 1980) as gate opening is more stimulus-driven. Task-related source-level theta band dynamics in all RIDE clusters were correlated with phasic pupil dynamics from 500 ms after stimulus onset. The finding that correlations between source-level theta band dynamics and pupil diameter were evident for all RIDE clusters suggests that the NE system modulates stimulus information, stimulus–response inhibition, and motor-response related information equally. This suggests that different informational contents coded in theta band dynamics are modulated simultaneously and that the degree of this modulation is similar for the different informational contents coded in the signal. It has been argued that the NE system modulates neural processes during task-relevant decision points (Aston-Jones and Cohen 2005). It is possible that in the first session, task-related decision processes during gate opening are strongly modulated by the NE system for all examined coding levels in the theta band dynamics. The opportunity cost model (Kurzban et al. 2013) suggests that effort, which is also reflected by the pupil diameter data and related to the NE system (Hopstaken et al. 2015), is modulated with time-on-task. The strong correlation between theta band dynamics and pupil diameter during WM gate opening in the first session suggests that the effort was primarily allocated in the WM gating opening control, and these processes were facilitated through phasic NE release, which enhances high-priority information (i.e., the updated reference) while suppressing the rest (Mather et al. 2016). In the last session, the pupil diameter became smaller, and task-related TBA decreased significantly. Moreover, also their correlation faded. This indicates that the investment of mental effort was gradually withdrawn with time or that the modulation of task-related decision processes during WM gate opening faded with time-on-task in prefrontal cortices. In addition, the pupil size diameter baselines gradually increased during the experiment, reflecting an enhanced tonic mode of NE systems (Gilzenrat et al. 2010). This increase in the tonic mode suggests that the overall perceived mental effort increased (Howells et al. 2010). Therefore, the high demand/cost in gate opening may lead to the withdrawal of effort, which was instead deployed to alternative tasks with lower opportunity cost (Brehm and Self 1989; Kurzban et al. 2013). This can also explain the dissociation between phasic pupil diameter and the task-related theta dynamics in the last session, suggesting that the NE activities and gate opening-related control processes became independent of each other under the effect of time-on-task.

## Conclusion

In conclusion, our study showed that WM gate opening, which requires more active control processes in the PFC, is more prone to time-on-task effect than WM gate closing processes. Based on the opportunity cost model (Kurzban et al. 2013), the performance decline of WM gate opening was likely because the high cost of gate opening control does not benefit in the long run; thus, the effort is allocated in alternative tasks. Our study also suggests that the NE system plays a critical role in this shift of effort allocation. In the early phase of WM gate opening, strong phasic NE release facilitates the prefrontal WM control processes. However, in the late phase, when the phasic NE activity wanes, its modulation on the cortical activities also fades with the increased disengagement on WM gate opening.

## Supplementary Material

Supplementary_materials_tgac001

## Data Availability

All data can be obtained from the corresponding author upon reasonable request.

## Notes

We thank all participants. *Conflict of Interest*: None declared.
