# Supplementary material for: Time-On-Task Effects on Working Memory Gating Processes—A Role of Theta Synchronization and the Norepinephrine System
Source: Cereb Cortex Commun. 2022 Jan 13;3(1):tgac001. doi: 10.1093/texcom/tgac001 (PMC8794645; doi:10.1093/texcom/tgac001)
Supplement: Supplementary_materials_tgac001 [file supplementary_materials_tgac001.zip › Supplementary_materials_tgac001.docx]

**Supplementary material**

**Time-on-task effects on working memory gating processes – a role of theta synchronization and the norepinephrine system**


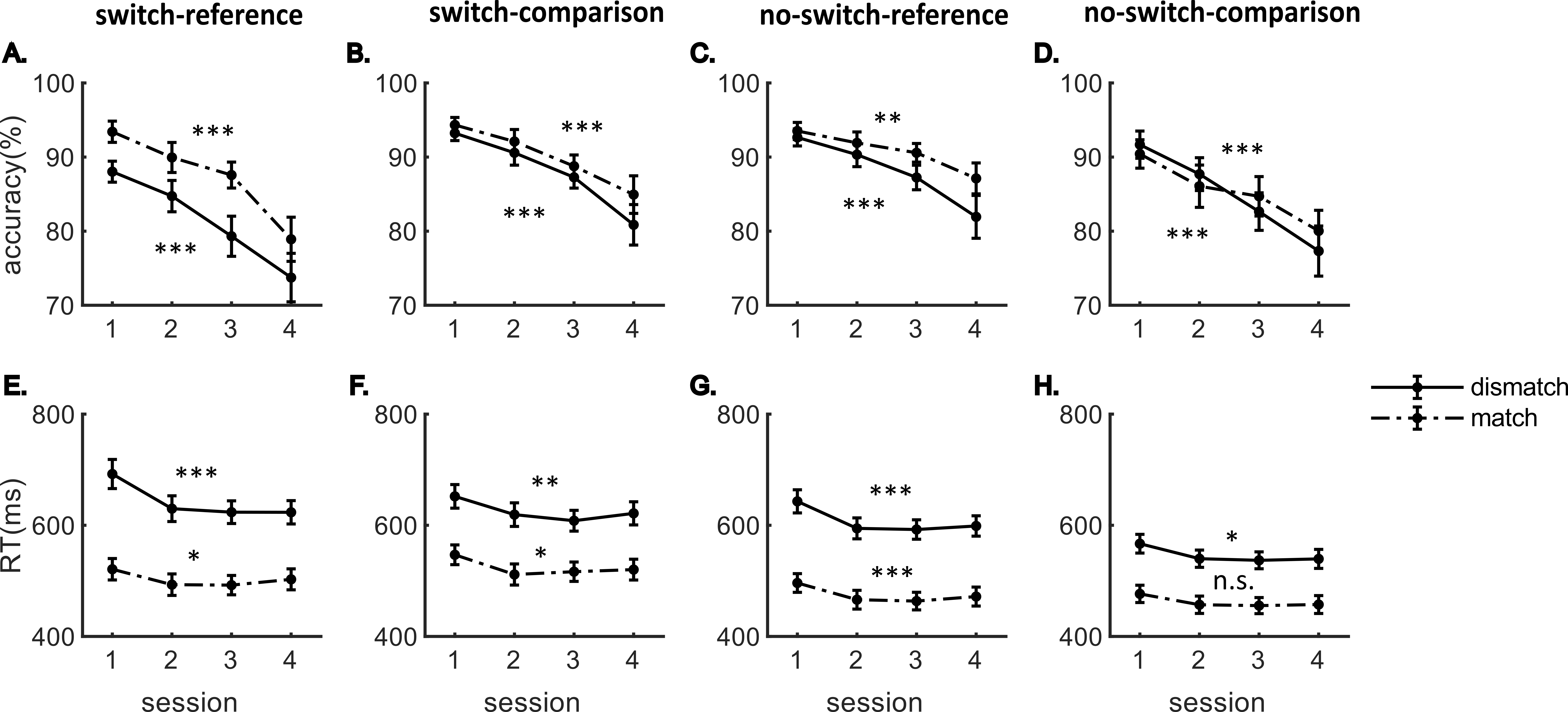


**Fig. S1** Behavioral performance for each condition. (A) to (D) shows the accuracies in each condition and session, (E) to (H) shows the corresponding RTs. . ‘*’, ‘**’, ‘***’, ‘n.s.’, represent significance value of p≤.05, p≤.01, p≤.005 and p>.05, respectively, calculated via repeated measures ANOVA using within-subject factor ‘session’ for each condition. Error bars indicate the standard error of mean. The accuracies significantly dropped from sessions S1 to S4 for all conditions (all F≥9.03, p≤.002). The repeated measures ANOVA using ‘RT’ also showed significant differences for most conditions (all F≥3.56, p≤.028) except for no-switch-comparison-match trials (F(1.80,54.04)=2.41, p=.11). RTs in all conditions generally declined from sessions S1 to S4.


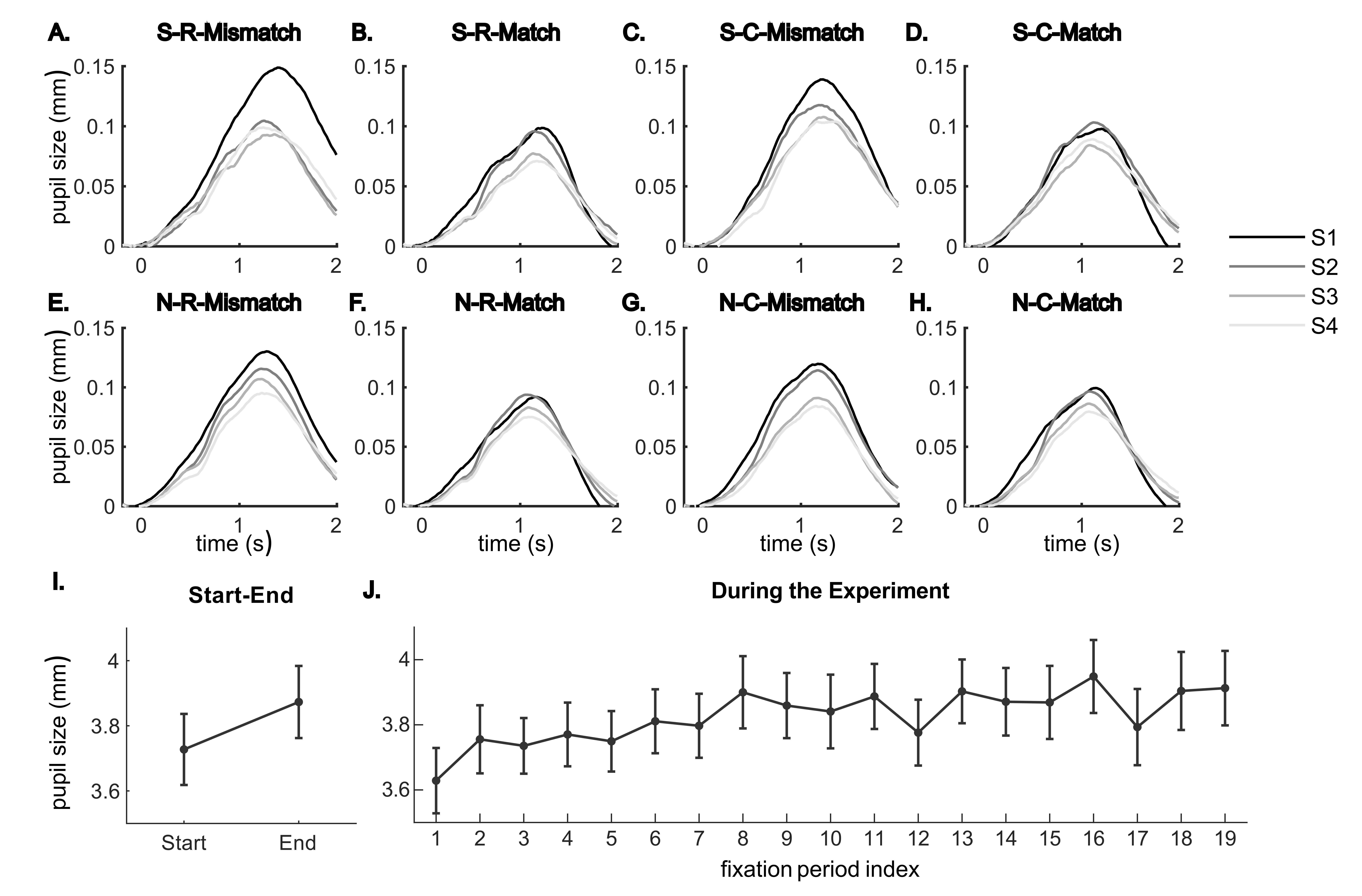


**Fig. S2** Pupil diameter for each condition and baselines. Plots (A) to (H) show the baseline-normalized pupil diameter for each condition. The condition is indicated by the title of each plot. ‘S’ means ‘switch’, ‘N’ means ‘no-switch’, ‘R’ means ‘reference’, ‘C’ means ‘comparison’, ‘Mis’ means ‘mismactch’, ‘Match’ means ‘match’. (I) and (J) represent the average pupil size during the resting status. Error bars indicate the standard error of mean. The absolute pupil size increased significantly from the start (3.73mm) to end (3.87mm) of the experiment (t(30)=2.21, p=.035, Cohen’ d=.40). A general increase on pupil baselines from 3.63mm to 3.91mm was also observed during the experiment and the repeated measures ANOVA revealed a significant difference among them (F(6.42,192.69)=2.44, p=.024).
